# Systemic Lupus Erythematosus and the Risk of Coeliac Disease: Substantial Discrepancies Among Studies Highlight a Significant Research Gap

**DOI:** 10.31138/mjr.101125.har

**Published:** 2026-09-01

**Authors:** Nektarios Marios Liapis, Arriana Gkouvi, Theodora Simopoulou, Dimitrios P. Bogdanos, Christina G. Katsiari

**Affiliations:** Department of Rheumatology and Clinical Immunology, Faculty of Medicine, School of Health Sciences, University of Thessaly, Biopolis, Larissa, Greece

**Keywords:** autoimmune disease, comorbidity, prevalence, serology, SLE

## Abstract

**Objective:**

Coeliac disease (CD) is an immune-mediated disorder that affects the small intestine. There are data showing that the prevalence of the disease is higher among patients with Systemic Lupus Erythematosus (SLE) compared to the general population. This narrative review investigates the correlation between these two entities.

**Methods:**

A thorough search was conducted in “PubMed,” “Scopus,” “Cochrane Central Register of Controlled Trials (CENTRAL),” “clinicaltrials.gov,” “gray literature” and “Google Scholar” from January 1, 1982, to July 1, 2025. A total of 2,991 articles were identified, of which 74 were reviewed in full text, and ultimately 15 were included in this review. Initially, the prevalence of biopsy verified CD among patients with SLE was examined. The prevalence of autoantibodies related to CD in patients with SLE was inspected subsequently.

**Results:**

The prevalence rate of both histologically confirmed CD and CD specific autoantibodies among patients with SLE exceeds the prevalence of CD found in the general population in most of the included studies, with noteworthy variability though.

**Conclusion:**

Based on the available data, patients with SLE appear to present greater risk of developing CD than the general population. However, the results between different studies vary significantly and routine screening for CD in patients with SLE is not currently recommended. The notable discrepancies among different studies underscore the necessity for further investigation to better understand the relationship between the two disorders.

## INTRODUCTION

Coeliac disease (CD) and systemic lupus erythematosus (SLE) are two clinical entities that have been extensively studied across all age groups. The prevalence of both appears to show an increasing trend on a global scale.^1^

CD is an immune-mediated disorder that primarily affects the small intestine precipitated by gluten ingestion.^2^ Among the most well-known genetic predisposing factors for CD are the human leukocyte antigen (HLA) genes HLA-DQ2 and HLA-DQ8, that play a key role in antigen presentation.^3^ From an immunological perspective, a compromised epithelial barrier with increased permeability allows gliadin, a component of gluten, to interact with T lymphocytes triggering an immune response.^4^ The prevalence of CD is considered to be between 0.5 and 2% among the general population and it mostly affects females.^5^ Clinical features are centred around malabsorption and include diarrhoea, weight loss, abdominal pain, iron deficiency anaemia, and ulcers.^6^ The European Society for the Study of Coeliac Disease (ESsCD) recommends that the diagnosis of CD is based on a combination of clinical presentation, serology and histopathological findings from small-bowel biopsy, while in selected cases a diagnosis can be made without a biopsy.^2^ Serology includes anti-tissue transglutaminase (tTG) antibodies which are the most sensitive, anti-endomysial (EMA) antibodies that are the most specific and anti-deamidated gliadin peptides (DGP). In contrast, anti-gliadin (AGA) antibodies demonstrate poor specificity.

SLE is a systemic autoimmune disease characterised by autoantibody production and immune complexes deposition, that can lead to multiorgan involvement and systemic inflammation.^7^ SLE predominantly affects females and the prevalence is around 0.1%, however this varies significantly between different regions.^8,9^ Gastrointestinal symptoms are common affecting approximately 50% of SLE patients, and often present with non-specific characteristics such as nausea, vomiting, anorexia and abdominal pain.^10,11^ GI symptoms in SLE can have multiple underlying causes such as disease activity, adverse effects of medications, infections and concomitant conditions such as CD.^11^

In recent years, there is growing evidence that patients with SLE may have a higher likelihood of developing CD compared to the general population. Additionally, overlapping clinical manifestations such as anaemia, nausea, fatigue, and other GI symptoms may obscure the diagnostic process, leading to the under-recognition of CD in patients with SLE. Two systematic reviews with meta-analyses published in 2024 examined the potential link between those two clinical entities.^9,12^ The significant discrepancies in prevalence rates reported across these meta-analyses reflect the heterogeneity of existing clinical studies and highlight an important research gap in this field. This paper makes an effort to address the existing gap by providing a narrative review of the studies that have examined the relationship between CD and SLE.

## MATERIALS AND METHODS

**Figure 1.**
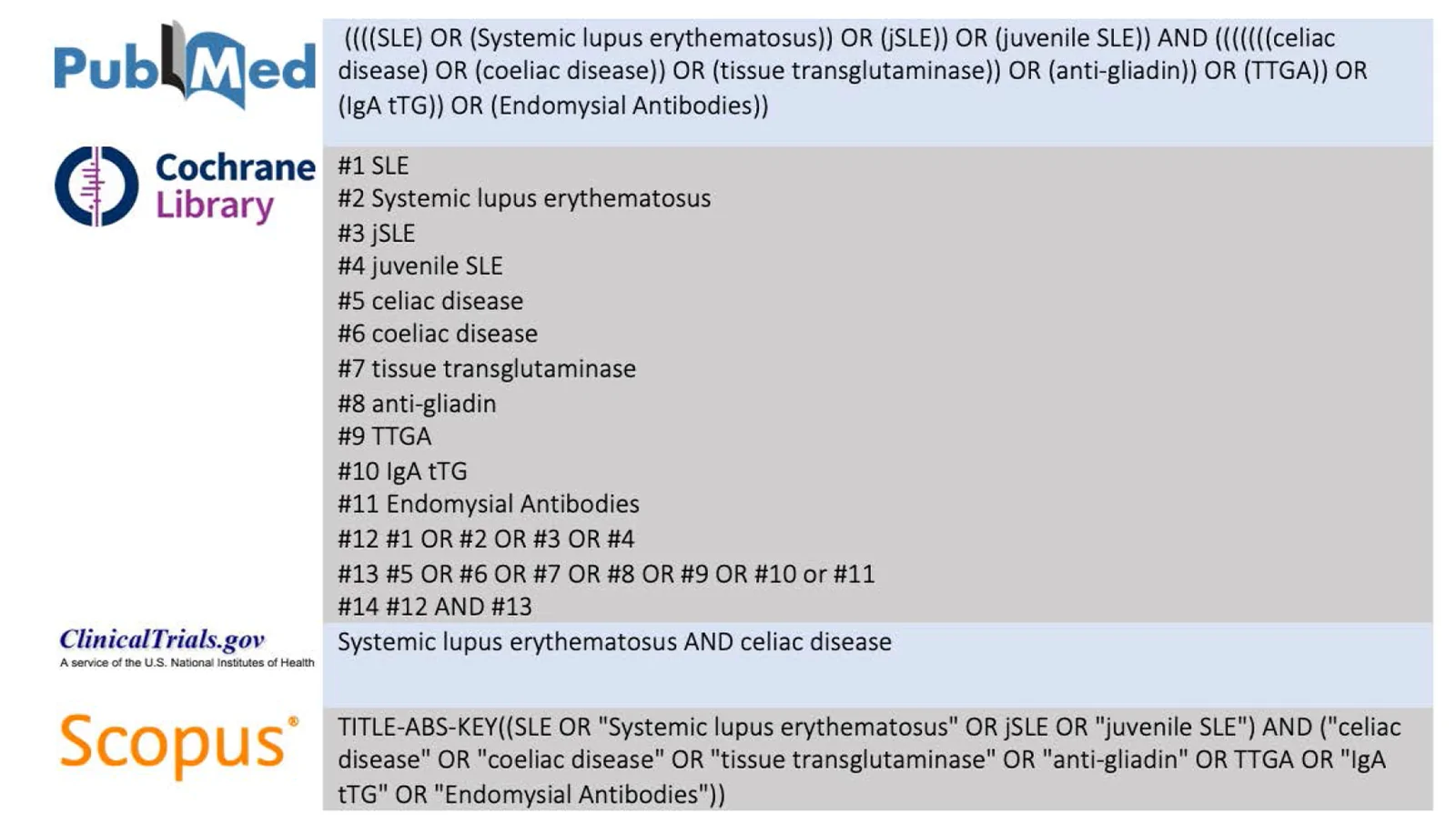
Search syntaxes used.

Information related to the current research was extracted from online databases “PubMed”, “Scopus”, “Cochrane Central Register of Controlled Trials (CENTRAL) “ and “clinicaltrials.gov”. Furthermore, gray literature and Google Scholar were also searched aiming to identify more relevant reports, as well as citation searching. Keywords included “systemic lupus erythematosus”, “SLE”, “juvenile SLE”, “celiac disease”, “tissue transglutaminase”, “anti-gliadin” and “endomysial antibodies”. Relevant synonyms and variants were also used, and search terms were adapted to each database (**Figure 1**). All types of articles from January 1, 1982, to July 1, 2025, that were relevant to our topic were studied (last search performed on 1 July 2025).

Emphasis was placed on bibliographic data from the last 10 years (2015 onwards). Two independent reviewers assessed the results. Each reviewer independently screened the titles and abstracts of the articles to identify those deemed appropriate for full-text evaluation. Disagreements regarding the inclusion of specific articles were resolved through discussion between the reviewers. In cases where this was not feasible, a third reviewer was consulted to arbitrate. The reference lists of the selected full-text articles were examined separately to identify any further relevant publications that may not have been retrieved through the search based on the keywords. The inclusion criteria were defined as follows:

1. Studies that examined the prevalence of histologically confirmed CD in patients with SLE
2. Studies that examined the prevalence of CD-specific autoantibodies in patients with SLE.

Initially, studies focusing on the prevalence of biopsy-confirmed CD in SLE population were assessed. Separately, studies mentioning the prevalence of autoanti-bodies associated with CD (anti-EMA IgA/IgG, anti-tTG IgA/IgG, AGA IgA/IgG, and anti-DGP IgA/IgG) in SLE patients were examined. Prevalence data for each country were extracted from epidemiological studies conducted in the general population at a certain timepoint, with particular attention to match the specific characteristics of the SLE population. For example, studies on childhood SLE were compared with epidemiological data in paediatric populations while also maintaining geographic alignment. However, it is mentioned that the prevalence rates within the general population depend significantly on the specific design and sample selection of each study. So, rates may vary across different studies and over time. No age and no language restrictions were applied. In addition to CD prevalence, we extracted data regarding study characteristics (first author, year, country, population type, gender and age group), number of patients, SLE classification criteria, SLE clinical characteristics (disease duration, activity and autoantibody profile where available), CD histologic grading (Marsh classification) and CD-related autoantibodies where available. Case reports, case series, letters to the editor, and studies that did not provide data relevant to the primary research question were excluded.

## RESULTS

### Included studies

**Figure 2.**
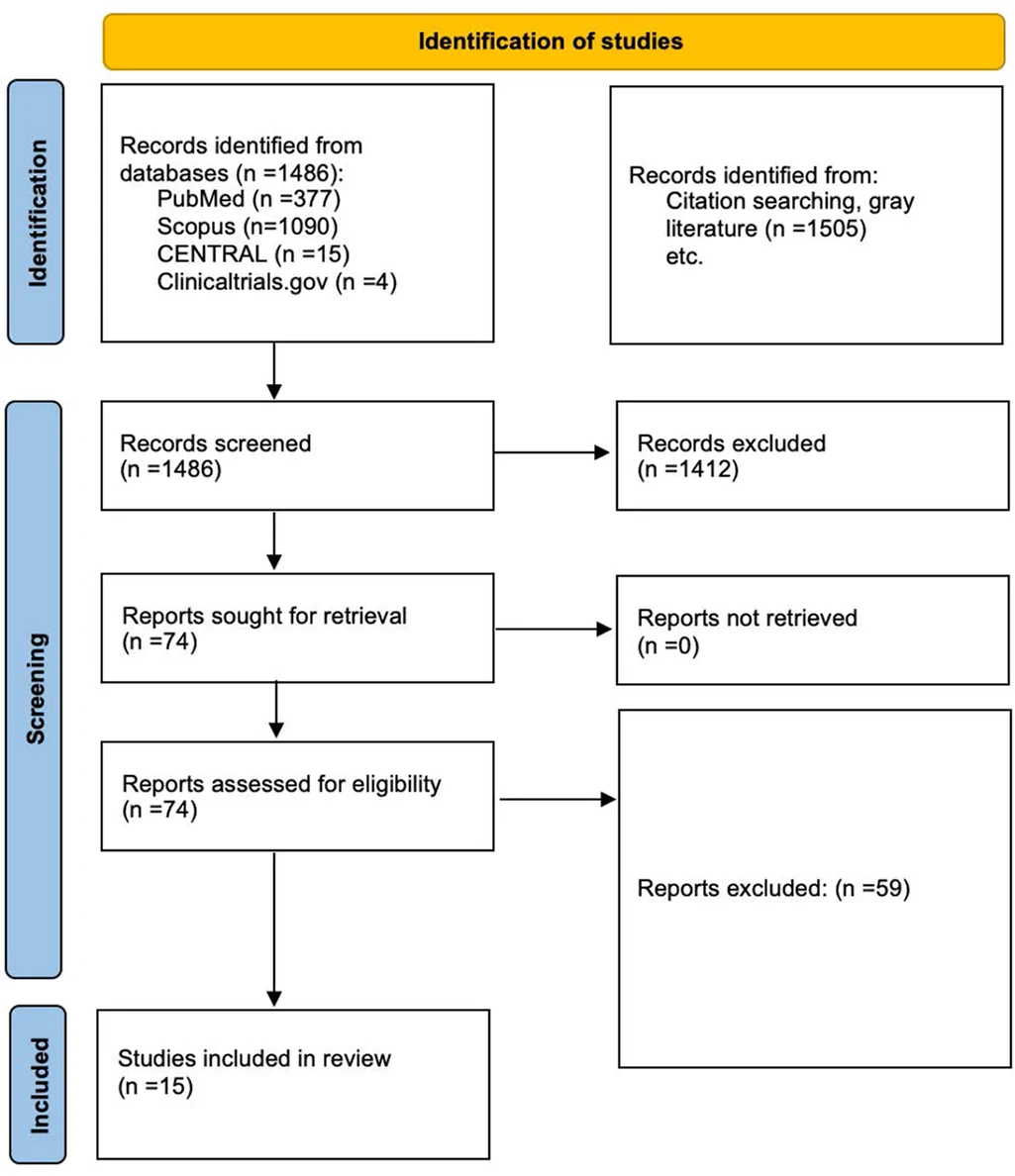
**(left).** Study selection flowchart.

Titles and abstracts from a total of 2,991 published articles were screened. Of these, 74 articles were deemed appropriate for full-text review. Upon further assessment, 15 articles finally met the inclusion criteria. The remaining 59 articles were excluded as they did not fulfil the inclusion criteria (**Figure 2**).

### Biopsy confirmed coeliac disease in SLE patients

**Figure 3.**
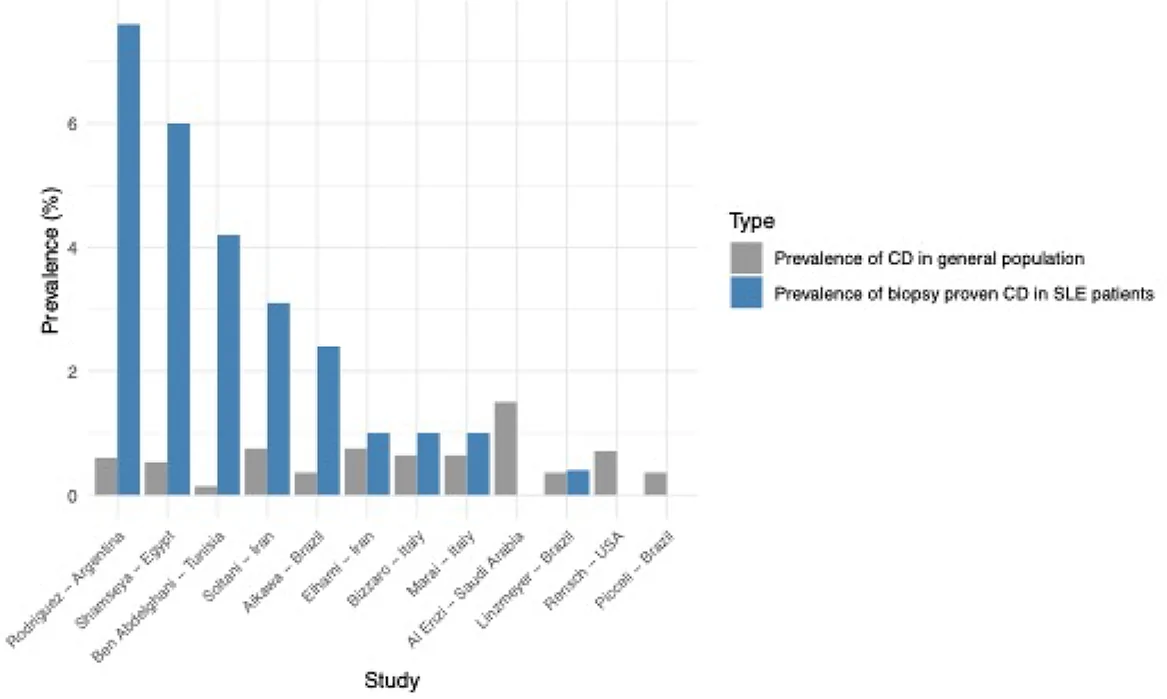
**(below).** Percentage of SLE patients with biopsy confirmed CD in relation to the prevalence of CD in the general population for each study.

In total, 12 articles provided information regarding biopsy proven CD (**Table 1**).^13–24^ The majority of the included studies (9 out of 12) reported higher prevalence of biopsy confirmed CD among SLE patients, compared with the prevalence of CD in the general population (**Figure 3**).^13,15–19,21,24^ The number of patients with SLE ranged from 24 to 281, while most studies utilised a sample consisting of more than 100 SLE patients.^14,15,18–24^ Furthermore, three studies did not report gender-specific data for SLE patients,^16,18,23^ yet across all available data and within each study, a female predominance was evident. Most studies were conducted in South America,^13,16,20,22^ followed by Asia and specifically Saudi Arabia and Iran.^14,15,19^ Europe was represented by two studies from Italy,^18,21^ Africa included Tunisia and Egypt,^17,24^ and finally one study came from the USA.^23^

**Table 1.** Biopsy-confirmed coeliac disease and related autoantibodies seropositivity in patients with systemic lupus erythematosus.

First author	Year	Country	Prevalence of CD in GP	N of SLE patients	Females % (N)	N of biopsies	Biopsy confirmed CD % (N)	Marsh classification	Seropositive % (N)
Aikawa^13^	2012	Brazil	0.36%^51^	41	85.4% (35)	1	2.4% (1)	No	2.4% (1)
Al Enzi^14^	2020	Saudi Arabia	1.50%^25^	115	90.4% (104)	13^*^	0% (0)	No	13.0% (15)
Ben Abdelghani^17^	2012	Tunisia	0.14%^26^	24	91.7% (22)	24	4.2% (1)	No	29.2% (7)
Bizzaro^18^	2003	Italy	0.64%^33^	100	NR	3	1.0% (1)	No	3.0% (3)
Caio^49^	2018	Italy	0.64%^33^	35	82.9 (29)	NA	NA	NA	8.6% (3)
Elhami^19^	2018	Iran	0.75%^#,52^	100	92.0% (92)	1	1.0% (1)	Yes	1.0% (1)
Gheita^29^	2012	Egypt	0.53%^53^	10	80.0% (8)	NA	NA	NA	60% (6)
Linzmeyer^20^	2020	Brazil	0.36%^51^	281	93.2% (262)	NR	0.4% (1)	No	NR
Marai^21^	2004	Italy	0.64%^33^	100	88.0% (88)	1	1.0% (1)	Yes	3.0% (3)
Picceli^22^	2013	Brazil	0.36%^51^	194	92.8% (180)	11	0% (0)	No	5.7% (11)
Rensch^23^	2001	USA	0.71%^54^	103	NR	24	0% (0)	No	23.3% (24)
Rodriguez^16^	2014	Argentina	0.60%^55^	26	NR	2	7.6% (2)	Yes	7.6% (2)
Sahin^$^,^31^	2019	Turkey	1.0%^30^	50	88.0% (44)	NA^$^	NA	NA	6.0% (3)
Shamseya^24^	2020	Egypt	0.53%^53^	100	90.0% (90)	10	6.0% (6)	Yes	10.0% (10)
Soltani^15^	2021	Iran	0.75%^#,52^	130	81.5% (106)	49	3.1% (4)	Yes	4.6% (6)^**^

CD: Celiac disease; GP: General population; N: Number; NA: Not applicable; NR: Not reported; SLE: Systemic lupus erythematosus.

*: Includes patients with rheumatoid arthritis, juvenile arthritis and SLE. Two patients declined biopsy. The study does not specify which subgroup these two patients belong to. So at least 13 patients with SLE underwent a biopsy.

**: Six patients tested positive for CD serological markers. According to the study methodology, patients were deemed appropriate for biopsy screening after evaluation by a gastroenterologist. Seropositivity was not necessary criterion.

#: 0.75% was estimated the percentage of biopsy verified CD in general population. Prevalence of CD in general population was 0.88% based on anti-tissue transglutaminase antibodies positivity.

$: Three patients were positive for tTG IgA antibodies. Patients were supposed to undergo a biopsy if positive EMA antibodies were identified. None of them had a positive result.

Among the 12 included studies, 9 of them reported cases of biopsy confirmed CD with a total of 18 cases. In the remaining 3 studies,^14,22,23^ although all seropositive patients underwent a biopsy (except for 2 who declined endoscopy), no biopsy confirmed CD cases were documented. It should be mentioned that although all 12 included studies defined the diagnosis of CD as biopsy proven, only 5 of them provided specific data regarding Marsh classification.^15,16,19,21,24^ It is further noted that the prevalence of histologically verified CD varied significantly across studies, ranging from 0% to 7.6%. Similarly, the prevalence of the disease in the general population differed by country, varying between 0.14% and 1.5%, and was not always proportionally reflected in the SLE patient subgroup (**Figure 3**). For instance, Saudi Arabia demonstrated the highest prevalence of CD in the general population (1.5%),^25^ yet the corresponding study reported 0 cases of biopsy-confirmed CD among SLE patients.^14^ In contrast, Tunisia, which had the lowest prevalence of CD in the general population (0.14%),^26^ showed the third highest rate of histologically verified CD among SLE patients (4.2%),^17^ following Argentina (7.6%)^16^ and Egypt (6.0%).24 Significant heterogeneity was also documented among the results of studies from different geographic regions. It is noted that in North America, represented by a single study including 103 patients from USA, no cases of histologically confirmed CD were identified.^23^

Regarding SLE classification, all studies utilised the American College of Rheumatology (ACR) classification criteria^27^ apart from two^14,24^ using the Systemic Lupus International Collaborating Clinics criteria (SLICC),^28^ while one group mentions a clinical diagnosis without specifying criteria.^23^ Three biopsy verified studies involved both children and adolescents.^13,14,24^ Further details regarding SLE-specific characteristics, CD, and SLE autoantibodies, disease duration and SLE activity can be found in **Table 2**. Furthermore, details regarding CD diagnosis in biopsy proven CD studies can be found in **Supplementary Table 1**.

**Table 2.** SLE and CD characteristics in all included studies.

**First author**	**SLE classification criteria**	**Population**	**Age**	**CD abs**	**SLE duration**	**SLE activity**	**SLE abs**
Aikawa^13^	ACR	Juvenile SLE	14.3 ± 3.8	IgA EMA	4.4 ± 3.7 yr	SLEDAI-2K = 8^$^	ANA 92.7%, anti-dsDNA 70.0%, anti-SM 26.8%, anti-RNP 22.0%, anti-Ro 34.1%, anti-La 14.6%, RF 9.8%
Al Enzi^14^	SLICC	N=81 adult SLE	34.1 ± 11.5 adults	IgA, IgG AGA, IgA EMA, tTG	NR	Mean SLEDAI = 9.0	NR
		N=34 childhood SLE	10.3 ± 2.7 children				
				AGA, EMA, tTG			
Ben Abdelghani^17^	ACR	Adults	36 (18–52)		NR	NR	NR
Bizzaro^18^	ACR	Adults	NR	IgA, IgG tTG	NR	NR	NR
Caio^49^	ACR	Adults	NR	IgA tTG, IgG DGP, IgA EMA	NR	NR	NR
Elhami^19^	ACR	Adults	44.9 ± 5.7	IgA tTG	NR	NR	NR
Gheita^29^	ACR	Children	12.2 ± 2.15	IgG, IgA tTG	1.3 ± 0.69 yr	Mean SLEDAI =12.8 ± 4.9	100% ANA, anti-dsDNA
Linzmeyer^20^	ACR	Adults	43 (34–53)	NR	36 mo (12–72 mo)	NR	NR
Marai^21^	ACR	Adults	Mean: 31 yr (Range: 15–56)	IgA, IgG tTG, IgA IgG EMA	NR	NR	NR
Picceli^22^	ACR	NR	Mean: 39 yr (Range: 17–67)	IgA EMA; all (+) were also tested for IgA tTG	NR	NR	ANA 99.4%, anti-DNA 30.6%, anti-Ro 35.0%, anti-La 20%, anti-SM 23%, anti-RNP 30%, aCL IgG 14.1%, aCL IgM 13%, LA 10%
Rensch^23^	NR	NR	NR	IgA and IgG AGA, EMA	NR	NR	NR
Rodriguez^16^	ACR	Adults	NR	tTG, EMA	NR	NR	NR
Sahin^31^	ACR	Childhood SLE	15.49 ± 3.39	IgA tTG, EMA		SLEDAI =13.5 ± 6.5	
Shamseya^24^	SLICC	Juvenile SLE	34.6 ± 9.6 current age; 11.9 ± 3.4 at diagnosis	IgA, IgG tTG; (+) were tested for EMA		SLEDAI =13.5 ± 6.5	NR
Soltani^15^	ACR	NR	31.5 ± 8.3 (range: 16–60)		4.6 ± 5.6	Mean SLEDAI = 2.5 ± 0.4	NR

Abs: antibodies; aCL: anti-cardiolipin antibodies; ACR: American College of Rheumatology; AGA: Anti-gliadin antibodies; ANA: Anti-nuclear antibodies; anti-dsDNA: anti-double stranded DNA antibodies; anti-RNP: anti-ribonucleoprotein antibodies; anti-Sm: anti-Smith antibodies; CD: Celiac disease; EMA: anti-endomysial antibodies; LA: Lupus anticoagulant; mo: months; NR: not reported; N: Number; RF: Rheumatoid factor; SLE: Systemic Lupus Erythematosus; SLEDAI: Systemic Lupus Erythematosus Disease Activity Index; SLICC: Systemic Lupus International Collaborating Clinics; tTG: anti-tissue transglutaminase antibodies; yr: years.

$: In 1 patient with CD.

### CD autoantibodies in SLE patients

The presence of CD-related autoantibodies was also investigated in the SLE population. All included studies reported higher prevalence of CD-related autoantibodies among SLE patients, compared with the prevalence of CD in the general population. Patients who tested positive for any of these antibodies were classified as seropositive. It was not feasible to determine with certainty the proportion of patients in each study who were found positive for each individual antibody, nor the antibody titters. CD seropositivity data were collected from 15 studies (**Table 1**). Like the histologically confirmed disease, considerable variability was observed among studies regarding the presence of CD-related autoantibodies, with prevalence ranging from 1% to 60%^19,29^ and not always following the general population disease rates. For instance, in Tunisia, where the general population prevalence of CD was the lowest (0.14%),^26^ 29.2% of SLE patients were seropositive for CD autoantibodies, the second highest percentage among the reviewed studies.^17^ In contrast, Turkey, which had relatively high general population CD prevalence (1%),^30^ showed relatively low rate of CD-related autoantibodies among SLE patients (6%).^31^

Based on the available data from all studies included in this review, biopsies were performed in a total of 79 SLE patients, who tested positive for at least one CD-related antibody. Of these, 17 had biopsies compatible with CD, representing 21.52% of the total. A special mention should be made of the study conducted in Tunisia, where all participants underwent biopsy irrespective of their CD antibody profile. This methodology enabled assessment of the predictive value of seropositivity for histologically confirmed CD. Based on this study, which included a small sample size of 24 patients, CD-related autoantibodies demonstrated a sensitivity of 14.28% and a specificity of 100% for histologically confirmed CD.^17^ Similarly, in the most recent of the two studies conducted in Iran, only six patients tested positive for CD autoantibodies in a total of 130 SLE patients. However, according to the study methodology, patients were considered suitable for biopsy screening following evaluation by a gastroenterologist, and seropositivity was not a necessary criterion. Consequently, a total of 49 patients underwent biopsy, of whom only six were seropositive, and four were confirmed to have biopsy verified CD. All four patients were also seropositive. In this context, the detection of CD-related autoantibodies indicated a sensitivity of 66.6% and a specificity of 100% for histologically confirmed CD.^15^ Interestingly, despite North America exhibiting one of the highest autoantibody prevalence rates (23.3%) among all studies, none of the patients had a histologically confirmed diagnosis of CD.^23^

### “CD” Diagnosis in SLE patients

It is worth mentioning two additional studies that were not included in the previous analyses due to insufficient information about diagnosis of CD and how it was confirmed. Nevertheless, they were considered noteworthy and are described here, primarily due to the large number of patients they involved. The first study, conducted in Israel, included 5,018 patients with SLE.^32^ The data were derived from Israel’s national healthcare system database. According to the study findings, 0.8% of these patients had a documented diagnosis of CD, which is notably higher than the estimated prevalence of 0.2% in the general Israeli population.^32^ The second study, conducted in Italy, examined 516 patients with a history of SLE who were being followed at a referral centre.^33^ It found that 1.72% of these patients had been diagnosed with CD, again a higher proportion than the estimated 0.6% prevalence of CD in the general population.

## DISCUSSION

This paper seeks to bridge the existing research gap regarding the true relationship between CD and SLE by offering a comprehensive review of the studies, examining the prevalence of both histologically confirmed CD and CD-specific autoantibodies among patients with SLE. Globally, the seroprevalence of CD is estimated at 1.4%, while CD prevalence for biopsy-confirmed cases at 0.7%.^34^ It is essential to recognise that the incidence of CD exhibits considerable variability related to age, sex, and geographic region. Based on the available data, it is plausible that SLE patients may have a higher likelihood of developing CD than the general population.

The underlying mechanisms linking these two conditions are complex and not yet fully understood. The HLA-B8 and HLA-DR3 genes appear to be common in both CD and SLE, potentially playing independent or collaborative roles in their pathogenesis.^35^ The triggering of the innate immunity, particularly via Toll-Like Receptors (TLRs) expressed on macrophages and dendritic cells, and the subsequent production of interferons, may also represent a shared pathophysiological pathway between the two diseases.^36,37^ Li et al. reported that clinically healthy individuals with high antinu-clear antibodies (ANA) titres exhibited upregulation of *TMG2*, which encodes the principal celiac autoantigen transglutaminase 2 (TG2) on whole blood gene expression profiling.^38^ While this does not indicate CD per se, it suggests that TG2-related pathways may be activated in a subset of ANA positive individuals, consistent with early, shared immune dysregulation that could be relevant to SLE-CD overlap.

A Mendelian randomisation study involving a population of 5,201 patients with SLE and 1,973 patients with CD from European ancestry, published in December 2023, presents particular interest.^39^ Studies like this aim to establish causality between exposure and outcome using single nucleotide polymorphisms (SNPs), which are DNA sequence variations that may influence gene expression and function. This approach is grounded in Mendel’s law of inheritance and has recently emerged as a powerful research tool. The study suggested that SLE (exposure) may significantly increase the risk of developing CD (outcome). Elevated risk was associated with the following SNPs: 1) rs389884 in the STK19 gene (encoding a serine/threonine kinase), 2) rs597808 in the ATXN2 gene (involved in endocytosis), 3) rs28361029 (not yet clearly characterised), 4) rs12524498 (also poorly described), and 5) rs6679677 in the PHTF1 gene.

To better clarify the association between CD and SLE, methodological parameters of the included studies must be considered. In everyday clinical practice, the diagnosis of CD relies on finding lesions that correspond to at least type 2 or 3 lesions in the Marsh classification.^40^ Undoubtedly, type 1 lesions pose a particular diagnostic problem as in many cases, they are interpreted ambiguously and generate diagnostic uncertainty. That is the reason why in many clinical studies, patients with a biopsy stage 1 are not classified as having CD. It is firmly believed that including these patients will probably lead to an overestimation of the true prevalence of the disease among the examined population. While all included studies in this paper labelled CD as histologically confirmed, only five provided clear staging data. Conversely, in two studies, a number of sero-positive patients were not biopsied and were classified as not having CD, resulting in possible underestimation of the disease prevalence. Significant differences were also observed in antibody testing methodologies. Most studies did not specify the positivity rates or titters for each antibody, nor the reference ranges used. AGA antibodies are known to be less specific than TTG and EMA.^41^ Furthermore, IgA deficiency testing was not always performed, despite its known impact on reducing serological sensitivity for CD diagnosis.^42^ These variables could either under- or over-estimate the true prevalence of the disease. The association between SLE and CD also operates in the reverse direction. In a large population-based Swedish cohort, Ludvigsson et al. reported an increased risk of SLE among individuals with biopsy verified CD although absolute risks remained low.^43^ This could be interpreted based on the assumption that GI complaints in patients without SLE may prompt a more extensive workup for primary GI disease including CD, whereas similar complaints in patients with established SLE may be attributed the disease or its treatment.

Moreover, the included studies span 2 decades from 2001 to 2021, during which diagnostic guidelines for both CD and SLE have evolved.^44,45^ Differences in age, ethnicity, genetics, comorbidities, disease activity, and SLE treatment further complicate interpretation. Only a few studies reported data on ongoing SLE therapy. Additionally, age at SLE onset may also contribute to variability in reported estimates. Of note, in the juvenile-onset SLE cohorts,^13,24,29,31^ CD seropositivity ranged widely between 2.4% to 60%, while biopsy confirmed CD was reported at 2.4% and 6%.^13,24^ In adult-onset SLE,^14,16–21,49^ CD seropositivity ranged from 1% to 29%, while biopsy confirmed CD was found between 0% and 7.6%. However, since no direct comparisons were made between juvenile SLE and SLE with adult onset, it remains unclear whether true CD prevalence differs between juvenile and adult-onset SLE. All these factors should be considered when analysing the outcome of each study. Geographic variations in the prevalence of biopsy-confirmed CD among SLE patients were also observed. Ethnicity is known to affect not only disease development but also prognosis in SLE. Patients from Africa, East and South Asia, and Latin America are more likely to develop severe forms of disease.^46^ Although heterogeneity in findings among different studies may partly reflect study design and population selection, it raises the concern of whether certain SLE subpopulations, particularly those with more severe disease, are more susceptible to develop CD. Additionally, the prevalence of CD-related autoantibodies does not mirror the geographic distribution of histologically confirmed disease. North America had among the highest sero-prevalence rates, yet no biopsy-confirmed cases were documented, based on a single U.S. study. While this may not adequately represent the entire region, the study’s large sample size warrants consideration. The prevalence of CD also differs widely across the general population. If SLE had no influence, the prevalence of CD in SLE patients would be expected to mirror general population rates. However, this is not the case. For example, although Saudi Arabia exhibits the highest prevalence rate of CD in the general population (1.5%),^25^ the proportion of patients with SLE who have histologically confirmed CD is 0. Therefore, according to this study, which included 115 individuals with SLE, these patients appear to present a lower risk of developing CD. Consequently, the question which arises is whether SLE acts as a protective factor against the development of CD in this case. In contrast, Tunisia, despite having the lowest prevalence rate of CD in the general population (0.14%),^26^ demonstrates the third highest percentage of histologically confirmed CD among SLE patients, amounting to 4.2%. Thus, according to the findings of this study, patients with SLE exhibit nearly a twenty-fold increased risk of developing CD compared to the general population. These two studies yield opposing findings despite geographic and ethnological proximity. Like biopsy-confirmed CD prevalence, CD autoantibody prevalence varies significantly across studies (1%–60%) without consistently correlating with general population prevalence rates of the disease. For instance, Turkey has a relatively high CD prevalence rate in the general population of 1% and Tunisia the lowest rate among the included studies of 0.14%.^26,30^ However, antibody positivity rates in the respective studies were 6%^31^ and 29.2%.17 Currently, no international guidelines recommend routine screening for CD in SLE patients except for those with suspicious clinical signs.^47^

Two systematic reviews with meta-analyses published in 2024 examined the potential link between CD and SLE. The first reported prevalence rate of biopsy-confirmed CD 0.7% and prevalence rate of CD specific autoantibodies 3.7% among SLE patients^9^ while the second reported 1.3% and 7.5%, respectively.^12^ The latter also examined the association of CD with primary Sjögren’s disease and systemic sclerosis, reporting significantly elevated prevalence of CD, particularly in Sjögren’s disease (5.6% for biopsy-confirmed CD and 10.4% for CD specific autoantibodies). Discrepancies in prevalence rates between the meta-analyses likely reflect methodological differences and sample selection criteria.

Diabetes mellitus type I and autoimmune thyroiditis are two diseases in which the prevalence of CD is significantly higher than that of the general population.^48^ There is also evidence supporting associations between CD and other autoimmune disorders. An Italian study found positive tTG-IgA antibodies in 3% of patients with a known history of an autoimmune disease, with the highest rate (5.8%) found in Sjögren’s disease.^49^ Similarly, another Italian study estimated an 8% prevalence rate of CD in systemic sclerosis.^50^ Specifically, 5 of 50 patients had positive tTG antibodies, and 4 of these had biopsy-confirmed CD. Overall, the above studies support the hypothesis that both CD and the associated autoantibodies are found more frequently in autoimmune diseases compared to the general population. However, more studies are needed in order to understand whether routine screening for CD should be recommended for patients with SLE. Our primary finding is the significant heterogeneity in study methodology and reported outcomes across existing studies. Although the prevalence of CD appears to be elevated in patients with SLE, notable geographic variation has been observed. Healthcare professionals should maintain a high index of suspicion for CD-related symptoms in patients with SLE. The recognition of such symptoms is not easy as the clinical manifestations of CD may overlap with those of SLE. Although CD is classically associated with diarrhoea, abdominal pain, and bloating, it can also present extraintestinal symptoms such as arthralgia or rash, features common in autoimmune diseases, including SLE. Approximately half of patients with SLE report gastrointestinal symptoms, potentially masking underlying CD and thereby delaying diagnosis.^15^

## CONCLUSIONS

Available data highlight a potential association between CD and SLE. Despite this evidence, significant discrepancies exist across studies regarding diagnostic approaches, population characteristics, and definitions of seropositivity. Due to this variability, routine screening for CD in SLE patients cannot be universally recommended at present. However, selective screening in patients, presenting with suggestive clinical features or additional risk factors for CD should be considered. Further investigation is necessary to better understand the relationship between the two conditions. Because geographical differences may reflect true variations due to different genetic background, regional guidelines for CD screening in SLE population may also be warranted. With sufficient evidence the development of an algorithm for early screening and intervention in SLE patients at increased risk of developing CD, could become feasible.

## SUPPLEMENTARY TABLES

https://mjrheum.org/assets/files/supplementary/MJR-2025-0402_Supp-Tables.pdf
